# Safety and efficacy of transpupillary silicone oil removal in combination with micro-incision phacoemulsification cataract surgery: comparison with 23-gauge approach

**DOI:** 10.1186/s12886-018-0878-z

**Published:** 2018-08-15

**Authors:** Wei Xu, Weijing Cheng, Hua Zhuang, Jian Guo, Guoxing Xu

**Affiliations:** Department of Ophthalmology, First Affiliated Hospital of Fujian Medical University, No. 20 Chazhong Road, Fuzhou, 350005 China

**Keywords:** Silicone oil removal, Micro-incision cataract surgery, Vitrectomy, Postoperative detachment, Endotamponade

## Abstract

**Background:**

To evaluate safety and efficacy of transpupillary silicone oil removal combined with micro-incision phacoemulsification cataract surgery, and to compare results of transpupillary with 23-gauge three-port vitrectomy approach.

**Methods:**

Consecutive cases that underwent silicone oil removal using either transpupillary or three-port approach in combination with micro-incision phacoemulsification cataract surgery were retrospectively reviewed. The main outcome measures were postoperative detachment rate, silicone oil residuals, best corrected visual acuity (BCVA) and intraocular pressure (IOP).

**Results:**

A total of 64 cases were included, 19 in transpupillary and 45 in three-port. Postoperative detachment rate within 3 months in transpupillary versus three-port was 15.8% versus 4.4% (*p* = 0.14), Silicone oil residuals was 7.4 ± 3.2% versus 7.1 ± 2.8% (transpupillary vs. three-port, *p* = 0.71). Preoperative versus postoperative BCVA (logMAR) was 1.49 ± 0.61 versus 1.42 ± 0.61 in transpupillary approach (*p* = 0.28) and 1.53 ± 0.48 versus 1.45 ± 0.57 in three-port approach (*p* = 0.11). Transpupillary approach resulted in lower IOP at postoperative day 2 (12.2 ± 2.3 mmHg vs. 13.5 ± 2.2 mmHg, *p* < 0.05), while postoperative follow-up at 1 month revealed no significant difference (*p* = 0.21).

**Conclusions:**

Transpupillary silicone oil removal combined with micro-incision phacoemulsification cataract surgery is less invasive and can be an alternative in some circumstances.

## Background

Silicone oil is frequently used as endotamponade in vitrectomy for some complicated cases such as giant retinal tears [1], traumatic endophthalmitis [2], and diabetic tractional detachment [3], which provides a clear view of the fundus in comparison with gas and inhibits postoperative vitreous hemorrhage [4]. However, complications including glaucoma, cataract, band keratopathy, emulsification of silicone oil and possible neural toxicity restrict it from a permanent vitreous substitute [5]. A number of vitreous substitutes have been investigated, but there are still barriers before clinical application [6]. Silicone oil removal is required according to post surgery follow-up. Attempt has been made to improve efficacy of and minimize surgical injury during silicone oil removal [7]. Potential complications should not be ignored. One of the most concerned complications due to silicone oil removal is postoperative retinal redetachment. Although efforts have been made to reduce percentage of detachment after silicone oil removal, the incidence reported varies from 3.5 to 13.2% [8, 9]. In the present study, we compared a transpupillary sclerotomy-free approach of silicone oil removal with standard three-port vitrectomy approach in terms of postoperative detachment rate, silicone oil residuals, visual acuity and intraocular pressure and so on.

## Methods

This retrospective study was approved by the Ethic Board of the First Affiliated Hospital of Fujian Medical University and complied with the tenets of the Declaration of Helsinki. Written informed consent was obtained from all individuals. Self-paid patients that underwent silicone oil-extraction combined with micro-incision phacoemulsification and intraocular lens implantation between May 2015 and April 2017 and had a minimum follow-up of 3 month were included. These patients previously underwent pars plana vitrectomy and silicone oil (Oxane® 5700; Bausch & Lomb, Rochester, USA) tamponade due to different incidence, while postoperative follow-up revealed fully attachment of retina and present of complicated cataract. Patients were fully informed regarding the difference between transpupillary approach and three-port vitrectomy approach to select a procedure at their own decision. The outcome measures were mean surgical duration, rate of postoperative detachment, silicone oil residuals in vitreous cavity, best corrected visual acuity (BCVA) and intraocular pressure (IOP).

In transpupillary approach, a standard phacoemulsification was performed and Viscoat® (Alcon, Fort Worth, USA) was injected into the anterior chamber followed by posterior capsulotomy. Infusion tip was plugged into corneal side incision and maintain irrigation height at 110 cm. Silicone oil was manually removed through an 18-gauge catheter system (BD, Suzhou, China) which was inserted directly into vitreous cavity via corneal main incision and posterior capsular hole. A 10 ml syringe was connected to the catheter system to actively aspirate silicone oil out. Intraocular lens was implanted into capsular bag followed by a gentle irrigation/aspiration step to remove Viscoat® and possible silicone oil residuals in the anterior chamber. Cases with intraocular lens implanted in sulcus were excluded.

In three-port vitrectomy approach, 23-gauge vitrectomy incisions were made after standard phacoemulsification and intraocular lens implantation. Infusion site was made in the inferotemporal quadrant, and intraocular manipulation sites were made in the superonasal and superotemporal quadrants for silicone oil extraction and illuminator. Silicone oil was actively removed under Stellaris™ PC machine (Bausch & Lomb, Rochester, USA). After extraction of the oil, retinal inspection was performed using Resight® viewing system (Carl Zeiss, Jena, Germany). Endolaser and epiretinal membrane peeling were performed when retinal break and epiretinal membrane were found.

Statistical analysis was performed using SPSS software for Windows version 16.0 (SPSS Inc., Chicago, IL, USA). Postoperative detachment rates between the two approaches were tested by Chi-square test. Visual acuity was converted to logMAR values, including hand motion and counting fingers as previously described [10]. Vitreous cavity was demarcated in binarized ultrasonic image. Silicone oil remnants were calculated and presented as percentage of total white area in the demarcated region using Image J. Descriptive statistics were presented as mean ± SD. The threshold for statistical significance was defined as *p*-value < 0.05.

## Results

A total of 64 consecutive cases were reviewed in this study. Demographic data of the patients was shown in Table 1. Gender ratios (male/female) were 10/9 in transpupillary approach and 23/22 in 23-gauge vitrectomy approach, respectively. The average age of the patients underwent silicone oil removal was 55.1 ± 7.0 years in transpupillary approach in contrast with 54.0 ± 11.9 years in 23-gauge approach. Among these cases, rhgematogenous retinal detachment is the most frequent cause that resulted in previous vitrectomy combined with silicone oil tamponade. The percentages were 63.2 and 77.8% (transpupillary vs. 23-gauge). Other causes include diabetic retinopathy, traumatic proliferative retinopathy and endophthalmitis. In the transpupillary group, patients had a mean duration of silicone oil tamponade for 8.0 ± 3.6 months, while in the 23-gauge group the mean duration was 6.5 ± 1.8 months.

**Table 1:** Demographic data of patients

	Transpupillary	23-gauge
Mean Age (years)	55.1 ± 7.0	54.0 ± 11.9
Gender		
Male	10	23
Female	9	22
Previous Diagnosis		
RRD	12 (63.2%)	35 (77.8%)
DRP	3 (15.8%)	6 (13.3%)
Traumatic PVR	3 (15.8)	3 (6.7%)
Endophthalmitis	1 (5.2%)	1 (2.2%)
Mean Duration of Tamponade (months)	8.0 ± 3.6	6.5 ± 1.8

*RRD* rhgematogenous retinal detachment, *DRP* diabetic retinopathy, *PVR* proliferative vitreous retinopathy

A significant difference in mean axial length was identified between transpupillary approach and 23-gauge approach, which was 24.5 ± 1.0 mm versus 23.9 ± 0.8 mm (*p* < 0.05). Transpupillary approach took shorter surgical duration than 23-gauge approach. The mean time required were 53.6 ± 8.2 min and 58.8 ± 9.5 min, respectively (*p* < 0.05, Table 2). In 23-gauge approach, 4 cases (8.9%) underwent endolaser coagulation and 3 cases (6.7%) underwent epiretinal membrane peeling. By contrast, these intraocular manipulations were infeasible in transpupillary approach. No significance in preoperative IOP was identified between transpupillary and 23-gauge method (13.2 ± 2.1 mmHg vs. 12.8 ± 1.8 mmHg, *p* = 0.14). Intraocular pressure at postoperative day 2 was lower in patients underwent transpupillary silicone oil removal than those using standard method (12.2 ± 2.3 mmHg vs. 13.5 ± 2.2 mmHg, *p* < 0.05). Postoperative follow-up revealed no significant difference in IOP between transpupillary and 23-gauge approach 1 month post-surgery (12.7 ± 3.4 mmHg vs. 13.8 ± 2.2 mmHg, *p* = 0.21). However, a consecutive record of postoperative IOP was not available.

**Table 2:** Clinical details and surgical outcomes between two approaches

	Transpupillary	23-gauge
Axial Length (mm)	24.5 ± 1.0^*^	23.9 ± 0.8
Surgical Duration (min)	53.6 ± 8.2^*^	58.8 ± 9.5
IOP (mmHg)		
Preoperative	13.2 ± 2.1	12.8 ± 1.8
POD2	12.2 ± 2.3^*^	13.5 ± 2.2
POM1	12.7 ± 3.4	13.8 ± 2.2
BCVA (LogMAR)		
Preoperative	1.49 ± 0.61	1.53 ± 0.48
Postoperative	1.42 ± 0.61	1.45 ± 0.57
Silicone oil residuals (%)	7.4 ± 3.2	7.1 ± 2.8

*IOP* intraocular pressure, *POD2* postoperative day 2, *POM1* postoperative month 1, *BCVA* best corrected visual acuity

^*^P < 0.05

**Fig. 1:**
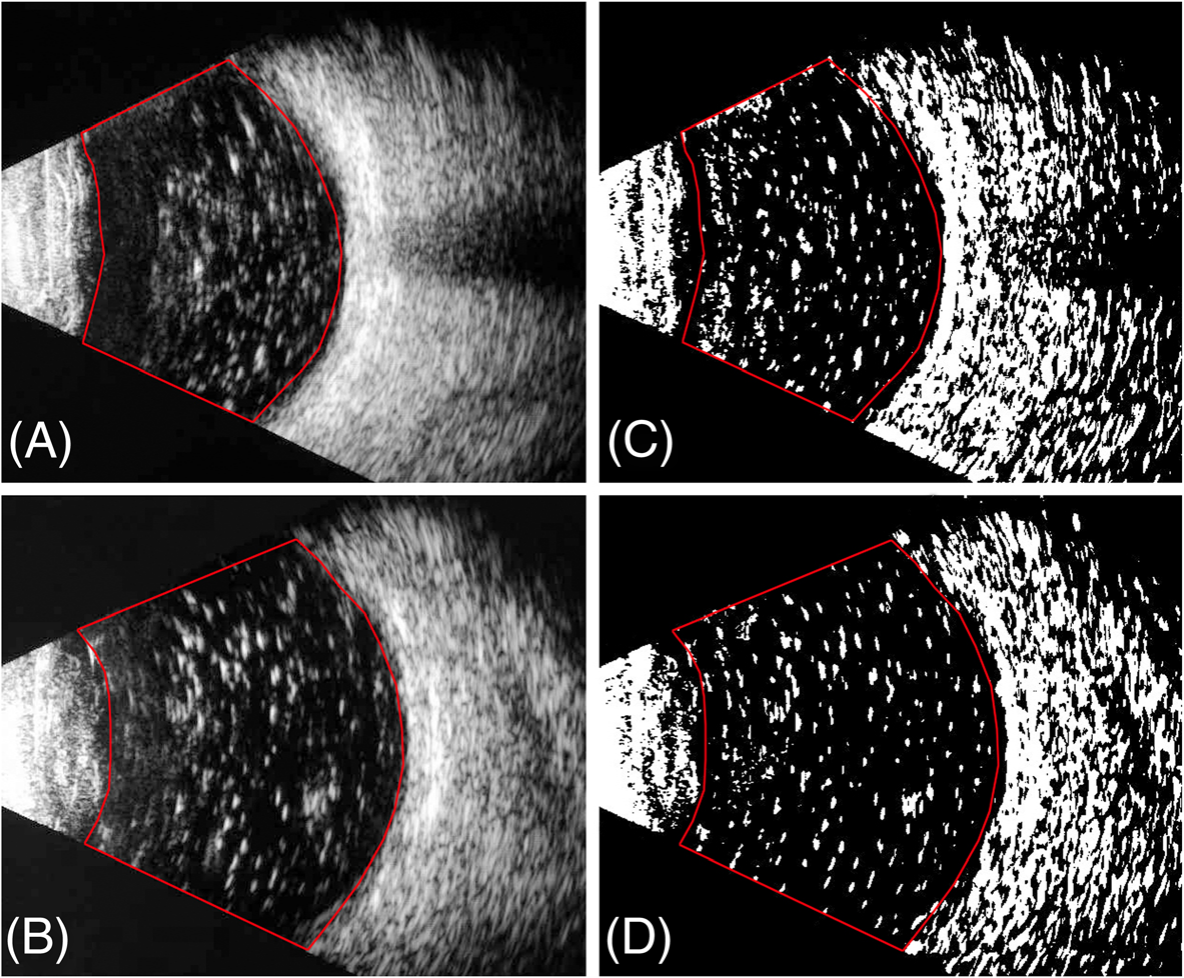
Postoperative silicone oil residuals. Ultrasonic image of Silicone oil residuals were magnified due to Rayleigh scattering. Representative images of patients who underwent silicone oil removal using transpupillary approach (**a**) and 23-gauge vitrectomy approach (**b**) were shown. Images were converted using a binarization method to quantify silicone oil remnants (**c** and **d**). The selected areas were analyzed and the results were presented as percentages

Three patients got detachment within 3 months in transpupillary approach. The postoperative detachment rate was 15.8%. By comparison, detachment within 3 months only occurred on two patients in 23-gauge approach. The postoperative detachment rate was 4.4%. However, statistical analysis failed to test significant difference regarding detachment rates between the two approaches (*p* = 0.14, Chi-Square test). Data of the postoperative retinal detachment cases were shown in Table 3. These patients underwent silicone oil reinjection. Anterior chamber silicone oil frequently appeared in one patient during follow-up. This patient previously underwent transpupillary silicone oil removal. Preoperative and postoperative BCVA were defined as the latest BCVA before silicone oil removal and BCVA at the last follow-up visit. Preoperative and postoperative BCVA in transpupillary approach were 1.49 ± 0.61 and 1.42 ± 0.61 without significance (*P* = 0.28). In 23-gauge approach the data was 1.53 ± 0.48 versus 1.45 ± 0.57, without significance either (*P* = 0.11). Silicone oil remnants were evaluated by ultrasonic image and appeared as percentages (Fig. 1). The percentages of silicone oil residuals in transpupillary versus 23-gauge approach were 7.4 ± 3.2% versus 7.1 ± 2.8% (*P* = 0.71).

**Table 3:** Details in cases of postoperative retinal detachment

Case	Previous diagnosis	Duration of tamponade (months)	Approach of removal	Endolaser or ERM peeling	Surgical duration (minutes)	Onset of postoperative detachment (weeks)
1	RRD	8	Transpupillary	No	55	2
2	RRD	6	Transpupillary	No	60	4
3	DRP	6	Transpupillary	No	61	8
4	RRD	3	23G	laser	56	5
5	RRD	5	23G	No	67	4

*RRD* rhgematogenous retinal detachment, *DRP* diabetic retinopathy, *PVR* proliferative vitreous retinopathy, *23G* 23-gauge three-port approach

## Discussion

Complications of silicone oil removal include recurrent retinal detachment, vitreous hemorrhage, postoperative hypotony, macular edema, etc. In this study, we compared the incidence of silicone oil removal related complications in two different removal approaches. Transpupillary silicone oil removal combined with cataract surgery has been reported previously with an infusion cannula connected to balanced salt solution [11]. But in this study we provide a sclerotomy-free way to extracted silicone oil with minimum injury in contrast to traditional three-port sclerotomy method. In addition, silicone oil removal from the anterior chamber allows administration of topical anesthesia which avoids the risk of eye ball perforation, retrobulbar hemorrhage and optic nerve injury. This less invasive procedure requires less intraoperative and postoperative monitoring, hence makes it an economical way [12]. The average cost for a patient undergoing silicone oil removal in this approach is 30% less than traditional three-port approach. However, Silicone oil removal using three-port sclerotomy enables intraoperative intervention against new retinal breaks or epiretinal membranes, resulting in a lower rate of postoperative detachment. The rate of retinal detachment after oil removal is much lower in the traditional three-port approach, although not significant statistically. It is worthwhile to note that sclerotomy introduces potential break in the peripheral retina which cannot be identified without scleral indentation. Irrigation fluid may penetrate into subretinal space through the iatrogenic break and develop postoperative retinal detachment.

When fluid penetrates through retinal breaks into subretinal space, liquid-filled vitreous cavity tends to maintain lower pressure. Lower IOP may induce vascular dilation or even rupture in retina and choroid. Therefore, postoperative hypotony is often related to vitreous hemorrhage and recurrent retinal detachment. Early postoperative hypotony is also a risk factor for choroidal detachment [13]. In this study, silicone oil removal from the anterior chamber resulted in relatively lower IOP early post-surgery. Postoperative detachment rate in transpupillary approach is higher compared with standard three-port approach, even though without statistical significance. We considered lower postoperative IOP as a potential risk for detachment, but the current data is not enough to illustrate a correlation between postoperative IOP and detachment rate. Interestingly, lower postoperative IOP is not linked to vitreous hemorrhage as no incidence existed in transpupillary approach.

Complicated cataract frequently develops in eye with silicone oil tamponade. A combined cataract surgery is often required before silicone oil removal. In some cases, posterior capsular opacity may impede silicone oil removal though scleral port. Hence posterior capsulotomy is required before a standard oil removal procedure. Silicone oil tends to flow into the anterior chamber in this circumstance. By comparison, transpupillary approach overcomes this weakness while providing an effective and less invasive way. Previous reported transpupillary silicone oil removal approaches have already concerned on efficiency and safety [14], but the incision was larger than what we did. In this study, we were able to remove silicone oil through limbal incision at a size suitable for micro-incision cataract surgery. Reduced incision will provide better visual outcome with minimum astigmatism. However, transpupillary method is only applicable to cases with complicated cataract. In cases with artificial or clear lens, traditional three-port method is required.

Surgical duration and silicone oil residuals in vitreous cavity are two indexes regarding the efficiency of a procedure. Although manual oil removal is administrated in transpupillary approach, it took shorter surgical duration than traditional three-port approach. Several factors account for this surgical time difference. In three-port technique, retinal inspection, laser coagulation and epiretinal membrane peeling took additional time. Besides, air-fluid exchange was used to ensure clearance of silicone oil, which also added to longer surgical duration. Interestingly, postoperative ultrasonic image did not reveal significant difference in silicone oil residuals between the two approaches. Silicone oil residuals appear much larger than they are in ultrasonic image due to Rayleigh scattering [15], which makes quantification of the residuals possible through ultrasonic imaging. Residuals after silicone oil removal were found positively correlated with axial length using this technique [16]. However, we did not find significant difference in silicone oil residuals between groups in our study, even though significant difference in axial length was identified.

The limitation of this study lies in that this is not a randomized study. Patients were able to select a procedure at their own decision after being well informed. Due to lower cost in the transpupillary approach, patients with financial concerning tend to select a cheaper procedure. Patients requesting the cheaper operation may have worse condition which may affect the results. Cases included in transpupillary approach were much less than in traditional three-port approach, which may also affect the interpretation of the results.

## Conclusion

In summary, transpupillary silicone oil removal combined with micro-incision cataract surgery is an effective and less invasive approach for the removal of silicone oil. But the infeasibility of intraoperative intervention on retina restricts selection of this surgical approach. Thoroughly preoperative inspection is required so as to reduce postoperative detachment.

## Abbreviations

BCVA: Best corrected visual acuity
DRP: Diabetic retinopathy
IOP: Intraocular pressure
PVR: Proliferative vitreous retinopathy
RRD: Rhgematogenous retinal detachment
